# Evaluation of antioxidant and cytoprotective activities of *Arnica montana* L. and *Artemisia absinthium* L. ethanolic extracts

**DOI:** 10.1186/1752-153X-6-97

**Published:** 2012-09-09

**Authors:** Oana Craciunescu, Daniel Constantin, Alexandra Gaspar, Liana Toma, Elena Utoiu, Lucia Moldovan

**Affiliations:** 1 Department of Cellular and Molecular Biology, National Institute of Research and Development for Biological Sciences, 296, Splaiul Independentei, 060031, Bucharest, Romania

**Keywords:** *A. montana*, *A. absinthium*, Antioxidant, Cytoprotective, Hydrogen Peroxide, Fibroblasts, Cell cycle

## Abstract

**Background:**

*Arnica montana* L. and *Artemisia absinthium* L. (Asteraceae) are medicinal plants native to temperate regions of Europe, including Romania, traditionally used for treatment of skin wounds, bruises and contusions. In the present study, *A. montana* and *A. absinthium* ethanolic extracts were evaluated for their chemical composition, antioxidant activity and protective effect against H_2_O_2_-induced oxidative stress in a mouse fibroblast-like NCTC cell line.

**Results:**

*A. absinthium* extract showed a higher antioxidant capacity than *A. montana* extract as Trolox equivalent antioxidant capacity, Oxygen radical absorbance capacity and 2,2-diphenyl-1-picrylhydrazyl free radical-scavenging activity, in correlation with its flavonoids and phenolic acids content. Both plant extracts had significant effects on the growth of NCTC cells in the range of 10–100 mg/L *A. montana* and 10–500 mg/L *A. absinthium*. They also protected fibroblast cells against hydrogen peroxide-induced oxidative damage, at the same doses. The best protection was observed in cell pre-treatment with 10 mg/L *A. montana* and 10–300 mg/L *A. absinthium*, respectively, as determined by Neutral red and lactate dehydrogenase assays. In addition, cell pre-treatment with plant extracts, at these concentrations, prevented morphological changes induced by hydrogen peroxide. Flow-cytometry analysis showed that pre-treatment with *A. montana* and *A. absinthium* extracts restored the proportion of cells in each phase of the cell cycle.

**Conclusions:**

*A. montana* and *A. absinthium* extracts, rich in flavonoids and phenolic acids, showed a good antioxidant activity and cytoprotective effect against oxidative damage in fibroblast-like cells. These results provide scientific support for the traditional use of *A. montana* and *A. absinthium* in treatment of skin disorders.

## Background

Aging, skin disorders, rheumatoid arthritis, atherosclerosis are caused by oxidative stress, occurring when production of reactive oxygen species (ROS) - in the form of hydroxyl radical, superoxide anion, peroxyl radical, singlet oxygen, hydrogen peroxide or ozone, exceeds the antioxidant protective capacity of target cells [1]. Free radicals are the initiators of a redox reaction cascade, resulting in changes of the chemical structure of biological macromolecules, such as proteins, lipids and DNA, or disturbances of human cell metabolism [2], or even tissue injury [3]. Human skin is exposed to both external factors, such as radiation, smoking, pollutants, organic solvents, pesticides [4] and internal ROS products from normal cell metabolism, normal aerobic respiration, stimulated polymorphonuclear leukocytes or macrophages [5,6], that increase the level of oxidative stress [7,8]. These conditions could delay the healing of skin injuries, such as burns, ulcers, wounds, eczema.

Synthetic and natural antioxidants are of particularly importance in maintaining the oxidative stress level under the critical point in human organism. Previous *in vitro*[9] and *in vivo*[10] studies reported the antioxidant capacity of several species of medicinal plants, acting at cellular level, through cell growth stimulation, membrane potential stabilizing or at molecular level, through ROS scavenging, lipid peroxidation, etc. [11]. These roles have been attributed, in part, to their biological active constituents, such as liposoluble and water-soluble vitamins (E and C, respectively) and polyphenolic substances [12].

*Arnica montana* L. (arnica) and *Artemisia absinthium* L. (wormwood) are two herbaceous perennial plants, native to temperate regions of Europe, including Romania. They belong to the same family, Asteraceae, subfamily Asteroideae, but *A. montana* is widely distributed in mountainous areas, while *A. absinthium* naturally grows at the edge of fields or rocky slopes. Traditionally, dried flowers of arnica are used, as tincture or ointment, for topically treatment of skin bruises, contusions and pain [13,14]. Aerial parts of wormwood, an aromatic herb, are used in liquid preparations, for treating skin wounds, boils, bumps or ulcers. Despite of their traditional use in skin disorders, no study has reported cytoprotective activity of arnica and wormwood extracts in a cell line of mouse fibroblasts.

The aim of this study was to investigate the chemical composition of both plant species, their antioxidant and fibroblast growth-stimulant activities. Polyphenolic composition of arnica and wormwood ethanolic extracts was analyzed by spectrophotometric assays and high performance liquid chromatography (HPLC). Their *in vitro* antiradical activity was investigated using Trolox equivalent antioxidant capacity (TEAC), Oxygen radical absorbance capacity (ORAC) and 2,2-diphenyl-1-picrylhydrazyl (DPPH) free radical assays. Protective effect of both plant extracts against hydrogen peroxide-induced oxidative stress in a mouse fibroblast-like NCTC cell line was evaluated by Neutral red and lactate dehydrogenase (LDH) methods, light microscopy and flow-cytometry.

## Results

### Chemical composition

Each plant was extracted in 70% (v/v) ethanolic solution, at room temperature, for 8 h, with extraction yields of 18.44% (w/w) for arnica flowers and 14.28% (w/w) for wormwood aerial parts (Table 1). The values of total phenolic acid and flavonoid compounds from both plant extracts are exhibited in Table 1. Wormwood extract had significant higher levels (p < 0.01) of total phenolic acids and flavonoids, in comparison with arnica extract.

**Table 1:** **Total phenolics content in *A. montana *L. and *A. absinthium *L. ethanolic extracts**

**Sample**	**Total phenolic compounds as caffeic equivalent (mg/g dry extract)**	**Total flavonoid compounds as quercetin equivalent (mg/g dry extract)**	**Extraction yield (%)**
*A. montana* L. extract	97.16 ± 1.37	38.62 ± 1.50	18.44 ± 0.76
*A. absinthium* L. extract	178.76 ± 1.58	52.43 ± 2.22	14.28 ± 0.69
Statistics	p < 0.01	p < 0.01	p < 0.01

All results were mean of triplicate measurements ± standard deviation. Paired t-test showed significant differences (p < 0.01) in total phenolic and flavonoid compounds between arnica and wormwood extracts.

HPLC analysis of arnica extract showed several peaks, among which 8 main peaks were prominent, at retention times of 2.232, 2.913, 14.386, 27.874, 28.919, 30.441, 31.416 and 32.443 min, while wormwood extract presented 3 prominent peaks at 14.319, 31.343 and 33.570 min (see Additional file 1). The compounds in plant extracts were identified by comparing them with reference standards of phenolic acids and flavonoids on the basis of their HPLC retention times (see Additional file 2). They were quantified by integration of the peak areas and the results are exhibited in Table 2. The content of each analyzed phenolic acid and flavonoid was significantly different (p < 0.01) between arnica and wormwood extracts. Arnica presented high levels of quercetin, rutin, apigenin and chlorogenic acid, with values of 1.881, 1.186, 0.501 and 0.329 mg/g dry extract, respectively, while wormwood had high levels of quercetin, luteolin, apigenin and caffeic acid, with values of 2.707, 0.677, 0.359 and 0.181 mg/g dry extract, respectively.

**Table 2:** **HPLC data of phenolic and flavonoid compounds content of *A. montana *and *A. absinthium *extracts**

**Compound (mg/gdry extract)**	***A. montana *L. extract **	***A absinthium *L. extract **	**Statistics**
Gallic acid	0.064 ± 0.003	0.092 ± 0.005	p < 0.01
Chlorogenic acid	0.329 ± 0.017	0.077 ± 0.004	p < 0.01
Caffeic acid	0.152 ± 0.008	0.181 ± 0.009	p < 0.01
Coumaric acid	0.038 ± 0.002	0.112 ± 0.006	p < 0.01
Ferulic acid	0.111 ± 0.006	0.100 ± 0.005	p < 0.01
Rutin	1.186 ± 0.058	0.089 ± 0.005	p < 0.01
Luteolin	0.077 ± 0.004	0.677 ± 0.036	p < 0.01
Quercetin	1.881 ± 0.101	2.707 ± 0.135	p < 0.01
Myricetin	0.011 ± 0.001	0.201 ± 0.011	p < 0.01
Apigenin	0.501 ± 0.027	0.359 ± 0.019	p < 0.01

Each value in the table is expressed in mg/g dry extract. All results were mean ± standard deviation (n = 3). Paired t-test revealed highly significant differences (p < 0.01) in each phytochemical, between arnica and wormwood extracts.

### Antioxidant activity

Antioxidant activity of ethanolic extracts of Romanian arnica and wormwood was determined by three complementary test systems: DPPH·, TEAC and ORAC assays. As shown in Table 3, both plant extracts exhibited significant activity towards scavenging of free radicals. The wormwood extract had higher values in TEAC and ORAC assays (690.62 and 917.89 μmol Trolox equivalents/g extract, respectively) than arnica extract (486.06 and 682.22 μmol Trolox equivalents/g extract, respectively), in correlation with its phenolic acids and flavonoids content. The IC_50_ value, calculated for DPPH free radical scavenging activity, decreased in the following order *A. montana* L. (0.63 ± 0.07 mg/mL) ) > *A. absinthium* L. (0.57 ± 0.05 mg/mL) > Trolox (0.28 ± 0.01 mg/mL). Trolox is a water-soluble derivative of vitamin E and was used as a standard due to its strong antioxidant activity.

**Table 3:** **Antioxidant activity values of *A. montana *L. and *A. absinthium *L. ethanolic extracts**

**Sample**	**Antioxidant activity**		
	**DPPH radical scavenging assay activity IC**_**50**_**(mg/mL)**	**Trolox Equivalent Antioxidant Capacity (μmol Trolox equivalents/g extract)**	**Oxygen Radical Absorbance Capacity (μmol Trolox equivalents/g extract)**
*A. montana* L. extract	0.63 ± 0.07	486.06 ± 20.63	682.22 ± 17.32
*A. absinthium* L. extract	0.57 ± 0.05	690.62 ± 13.79	917.89 ± 15.83
Statistics	p < 0.01	p < 0.01	p < 0.01

All results were mean of triplicate measurements ± standard deviation. Paired t-test showed significant differences (p < 0.01) in antioxidant activity between arnica and wormwood extracts.

These results showed that wormwood extract exhibited 1.4-fold higher antioxidant capacity than arnica extract, which correlated with wormwood amount of phenolic acids found to be 1.8-fold higher and the amount of flavonoids about 1.4-fold higher than those in arnica extract.

### Influence of plant extracts on cell viability

**Figure 1:**
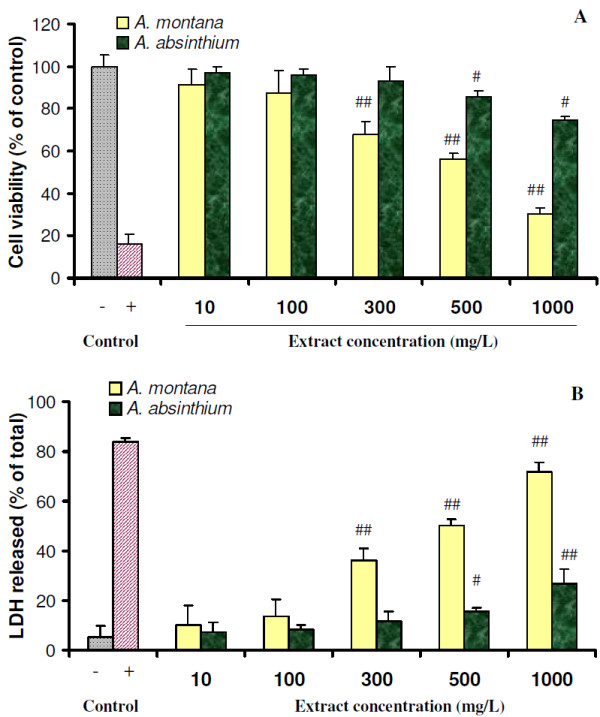
**Cell viability of NCTC cells cultured with different concentrations of * A. montana *L. and * A. absinthium *L. extracts, analyzed by Neutral red (A) and LDH (B) assays.** The negative control was represented by cells cultivated in culture plate, in MEM (dotted) and the positive control was represented by cells cultivated in MEM containing 100 μM H_2_O_2_ (striped). Results are shown as mean ± SD (n = 6). Pairs of negative control and each sample were analyzed by t-test. Significant differences in each pair are marked with ^#^p < 0.05 or ^##^p < 0.01.

To exclude the possible cytotoxic effect of of arnica and wormwood extracts, several concentrations (10–1000 mg/L) were tested on NCTC cells. As shown in Figure 1, cell treatment with each concentration of plant extract, for 24 h, induced a dose-dependent effect on cell viability values. Higher values of cell viability (above 80%) were recorded for concentrations up to 100 mg/L of arnica extract and up to 500 mg/L of wormwood extract (Figure 1A). Higher concentrations induced disturbance of Neutral red retention and cell viability significantly decreased (p < 0.01) down to 30.46%, compared with negative control (100%) (Figure 1A).

We also examined the presence of LDH cytosolic enzyme in the cell culture medium as an indicative of cell membrane damage. Cells treated with 10–100 mg/L arnica extract and 10–500 mg/L wormwood extract secreted less than 15% LDH into the culture medium (Figure 1B). Higher concentrations of plant extracts resulted in cell damage and the value of released LDH increased up to 71.7%, significantly higher (p < 0.01) than negative control (5.5%) (Figure 1B).

This data allowed selecting the optimal range of non-cytotoxic concentrations of each plant extract (up to 100 mg/L arnica and 500 mg/L wormwood), used in further experiments.

### Effect of plant extracts on H_2_O_2_-induced oxidative stress in NCTC cells

H_2_O_2_, a precursor of various ROS, was chosen as oxidant reagent in this study. Various concentrations of H_2_O_2_ (20–500 μM) were used to determine the appropriate dose. The dose of 50 μM H_2_O_2_ that reduced the fibroblast viability at approx. 50% was used in further experiments. Higher doses decreased dramatically the cell viability down to 11% at 500 μM H_2_O_2_.

**Figure 2:**
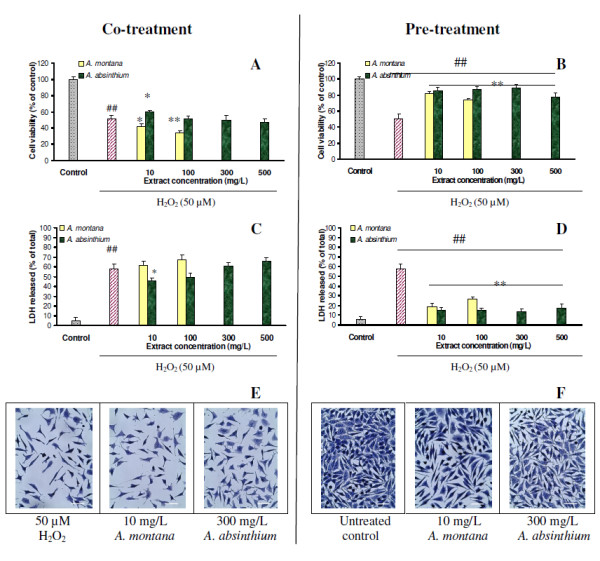
**Viability of NCTC cells after co-treatment (A, C) and pre-treatment (B, D) with plant extracts at various concentrations, analyzed by Neutral red (A, B) and LDH (C, D) assays.** Results are represented as mean ± SD (n = 6). ^##^p < 0.01 compared with untreated control (dotted); *p < 0.05 and **p < 0.01 compared with H_2_O_2_-treated group (striped). Micrographs taken at 24 h after H_2_O_2_-treatment showed the morphology of cells after co-treatment (**E**) and pre-treatment (**F**) with 10 mg/L *A. montana* extract and 300 mg/L *A. absinthium* extract. Scale bar = 10 μm.

In order to evaluate the ability of both plant extracts to reduce oxidative stress in H_2_O_2_-treated cells, co-treatment and pre-treatment experiments were done and results are presented in Figure 2. Cell viability, membrane integrity, morphology and cell cycle were investigated. *Co-treatment* with 10–100 mg/L arnica extract or 100–500 mg/L wormwood extract, simultaneously with H_2_O_2_, presented viability values similar to H_2_O_2_-treated group (Figure 2A). Only the cells treated with 10 mg/L wormwood presented a significantly (p < 0.05) higher value of cell viability (approx. 60%) compared to H_2_O_2_-treated group (Figure 2A). LDH release readings for arnica and wormwood extract co-treatment groups did not vary significantly from H_2_O_2_-treated group (p > 0.05), with the exception of the 10 mg/L wormwood group that was significantly lower (p < 0.05) (Figure 2C). In comparison, *pre-treatment* of NCTC cells with arnica and wormwood extract, at each tested concentration, significantly reversed the H_2_O_2_-induced cytotoxicity. Neutral red test results presented values between 75–88.9% cell viability, significantly (p < 0.01) higher than H_2_O_2_-treated group (Figure 2B). The best cell protection, expressed as cell viability values, was observed for pre-treatment with 10 mg/L arnica and 10–300 mg/L wormwood, respectively. LDH secretion in the culture medium was significantly (p < 0.01) reduced when NCTC cells were pretreated with each concentration of arnica and wormwood extracts, compared to H_2_O_2_-treated group (Figure 2D).

Morphological analysis was carried out for cells co-treated and pre-treated with each concentration of plant extracts and 50 μM H_2_O_2_. H_2_O_2_-treated cells appeared degenerated and lysed (Figure 2E, left). Similarly, cells co-treated with plant extracts and H_2_O_2_ presented an altered cell morphology and decreased cell density, indicating a damaging effect, regardless of the extract concentration (Figure 2E, middle and right). On the other hand, untreated cells (Figure 2F, left) and cells pre-treated with 10 mg/L arnica and up to 300 mg/L wormwood extract concentrations did not show modified morphology (Figure 2F, middle and right). Cells were homogeneously distributed on the plate and exhibited the typical spindle shape morphology of normal fibroblasts. Some cells in mitosis and no apoptotic bodies were seen on the pretreated plates, similar to the untreated group.

**Figure 3:**
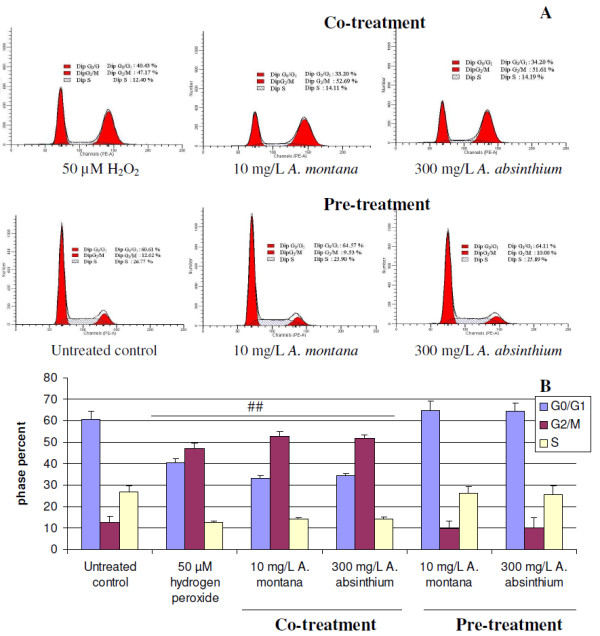
**A Effect of plant extracts on cell cycle distribution of NCTC cells.** Co-treated and pre-treated cells were stained with propidium iodide and analyzed by flow-cytometry. **B** DNA histograms analyzed by MODFIT^TM^ LT 3.0 software. Results are represented as mean ± SD (n = 6). Each phase of individual samples was compared with correspondent phase in untreated control using paired t-test. Statistically significant differences were observed (^##^p < 0.01).

Cell cycle distribution was analyzed after treatment of H_2_O_2_-injured cells with 10 mg/L arnica and 300 mg/L wormwood, respectively. As shown in Figure 3A, H_2_O_2_-treated cells had an altered cycle that was arrested in G0/G1 and G2/M phases. The results showed a proportion of cells in G0/G1 phase that decreased from 60.61% in untreated control to 40% in H_2_O_2_-treated cells and an increase of cells in G2/M phase, from 12.62% in untreated control to 47% in injured cells (Figure 3B).

***Co-treatment*** of NCTC cells with plant extracts and hydrogen peroxide resulted in distribution of cell cycle events similar to H_2_O_2_-treated cells (Figure 3A) and values of DNA content were significantly different (p < 0.01) from the untreated control (Figure 3B). Co-treatment with arnica and wormwood extracts indicated values of 33.20% and 34.20% DNA in G0/G1 phase, respectively, and 52.69% and 51.61% DNA in G2/M phase, respectively, close to those of H_2_O_2_-treated cells. In addition, the proportion of cells in S phase decreased in co-treatment to values similar to H_2_O_2_-treated cells (12.40%) (Figure 3A, B).

In contrast, ***pre-treatment*** of NCTC cells with plant extracts significantly reversed the effect induced by H_2_O_2_ in cell cycle distribution (Figure 3A). DNA content in each phase was not significantly different (p > 0.05) from untreated control (60.61% in G0/G1 phase, 12.62% in G2/M phase and 26.77% in S phase) (Figure 3B). These values indicated the protective role of arnica and wormwood extracts in pre-treated NCTC cells.

All these results obtained in cell culture experiments showed that pre-treatment with 10 mg/L arnica and 10–300 mg/L wormwood presented the best protective effect against H_2_O_2_-induced oxidative stress.

## Discussion

Many previous studies have investigated the relationship between the antioxidant activity of plant products and their polyphenolic content [15-17]. Polyphenolic compounds are very important antioxidants due to their hydroxyl groups that confer free radical scavenging ability to plant products. Several methods are performed to evaluate the antioxidant activity of different plant products, due to the complex nature of phytochemicals [18]. There are methods, such as ORAC, based on hydrogen atom transfer, in which the antioxidant and the substrate compete for thermally generated peroxyl radicals through decomposition of azo-compounds [19]. The methods based on electron transfer, such as TEAC, measure the capacity of an antioxidant to reduce an oxidant that changes its colour when reduced, proportionally to the antioxidant concentration [20]. Free radical DPPH scavenging assay is based on a reaction mechanism of both electron and hydrogen atom transfer [21]. In our study, ethanolic extracts of arnica and wormwood, rich in phenolic acids and flavonoids, showed good free radical scavenging activity by TEAC, ORAC and DPPH assays. A linear correlation between their polyphenolic content and ORAC, TEAC antioxidant activities was previously demonstrated [22]. This is supported by an earlier study showing that wormwood antiradical activity, assayed by electron spin resonance spectroscopy, correlated well with its high content of polyphenolic compounds [23]. The extent of plant extracts antioxidant activity must be further analyzed using assays based on other action mechanisms, such as inhibition of enzymes and chelating trace elements involved in free-radical production [24,25].

The plants studied in the present work are used to treat a variety of diseases in traditional medicine. *A. montana* L. is used for external treatment of skin lesions, eczema or tendon and joint inflammation [26]. *A. absinthium* L. is external used in wound healing and eczema and for internal treatment of abdominal pain and hemorrhoids [27]. Production of ROS in excess is responsible for several diseases including skin disorders. Pharmacological agents having modulator effect in oxidative stress processes can be monitored at cellular level. In the present study, an *in vitro* experimental protocol was designed to injure fibroblast-like cells, using hydrogen peroxide as oxidant reagent. Being an unstable and highly reactive compound, hydrogen peroxide is toxic to cells [28] and known to induce senescence-like growth arrest in human diploid fibroblasts [29]. According to other authors, low concentrations of hydrogen peroxide preferentially induced cell death through apoptosis [30].

Ethanolic extracts of *A. montana* and *A. absinthium* were tested for their ability to reverse H_2_O_2_ effect in injured NCTC cells using co-treatment and pre-treatment protocols. We used the vital stain Neutral red and LDH test to assess the effect of plant extracts on cell viability and also light microscopy for cell morphology observations and flow-cytometry for cell cycle analysis. Several studies used 3-(4,5-dimethylthiazol-2-yl)-2,5-diphenyltetrazolium bromide (MTT) for cell viability evaluation, but Bruggisser et al. [31] showed that phenolics from plant extracts reacted with MTT and a direct reduction to formazan took place in the absence of cells. Plant extract pre-treatment induced a 1.7 to 2-fold increase of cell viability, assayed by Neutral red test, compared to corresponding plant extract co-treatment. The cytosolic enzyme LDH is an *in vitro* marker for membrane integrity [32]. The results of LDH released after arnica and wormwood pre-treatment of NCTC cells were 1.35 to 1.6-fold, respectively, lower than in the co-treatment experiment, indicating their potential application in the treatment of ROS-mediated cell membrane damage. Cell morphological observations demonstrated that both plant extracts were able to protect mouse fibroblasts from oxidative stress when they were added 24 h before H_2_O_2_ insult (Figure 2F).

The control of the cell cycle is a highly regulated process that involves a complex cascade of events [33]. It is assumed that H_2_O_2_ could induce the stop of cell cycle in G2/M phase, through expression of a cyclin-dependent kinase inhibitor [34]. In the present study, cells exposed to H_2_O_2_ suffered a partial blocking in G2/M phase caused by DNA damage (Figure 3). Cells co-treated with plant extracts and H_2_O_2_ presented an increase of the cell proportion in G2/M phase. It was also observed an increase of cell proportion in S phase, which could be due to a mitochondrial DNA damage in fibroblast cells [35]. Our results indicated that co-treated NCTC cells suffered oxidative damage and were blocked in the current phase of the cell cycle, similar to H_2_O_2_-treated cells. It could be also suggested that co-treatment induced an increase of ROS production and cell damage. A very interesting result of this study was registered during cell cycle analysis of NCTC cells pre-treated with each plant extract. The data demonstrated that *A. montana* and *A. absinthium* extracts protected NCTC cells against oxidative damage, supported cell proliferation and showed no alterations in cell cycle caused by H_2_O_2_-induced oxidative stress.

## Experimental

### Chemicals

A cell line of mouse fibroblasts (NCTC clone 929) was obtained from the European Collection of Cell Cultures (Sigma-Aldrich, USA) and all the materials for the experiments involving cultured cells were purchased from Sigma-Aldrich (St. Louis, MO, USA). CytoTox 96 kit for lactate dehydrogenase (LDH) activity and RN-ase were from Promega (USA). Propidium iodide (PI) was purchased from BD Biosciences Pharmingen (San Diego, CA, USA). 2,2-diphenyl-1-picrylhydrazyl (DPPH), hydrogen peroxide (H_2_O_2_), 6-hydroxy-2,5,7,8-tetramethylchromane-2-carboxilic acid (Trolox), Neutral red (NR) were purchased from Merck (Germany). HPLC grade methanol, acetonitrile, gallic acid, chlorogenic acid, caffeic acid, p-coumaric acid, ferulic acid, rutin, luteolin, quercetin, myricetin and apigenin, were purchased from Sigma-Aldrich (USA). All other reagents for biochemical analyses with high analytical grade were from Merck (Germany).

### Preparation of plant extracts

The plant materials used in the research were flowers of *A. montana* L. and aerial parts of *A. absinthium* L., species from Cluj county, Romania. They were identified and authenticated by Dr. G. Coldea, Department of Taxonomy and Ecology, Institute of Biological Research Cluj-Napoca, Romania. Voucher specimens of *A. montana* L. (No. 647621) and *A. absinthium* L. (No. 637250) were deposited at Herb, Botanical Garden Cluj-Napoca, Romania. Plants were air-dried, at room temperature, in the absence of light and finally powdered. Dried materials (75 g) were extracted in ethanol/water (70/30, v/v), in a ratio material:solvent of 1:10 (w/v), on a shaker (200 rpm), at room temperature, for 8 h. The extracts were separated from the material residue by filtration through Whatman No. 1 filter paper. The solvent was removed by evaporation to dryness, *in vacuo*, at 40°C, using a rotary evaporator (Heidolph VV Micro, Germany) and, finally, the extracts were freeze-dried in a lyophilizer Christ Gamma 1–16 LSC (Germany). The dry extracts were weighed and stored at −20°C until used. The percentage yield was expressed in terms of dried weight of plant material. For cell culture experiments, the dry extracts were dissolved in phosphate buffered saline (PBS), pH 7.4.

### Determination of total phenolic and flavonoid content

Total phenolic content of each plant extract was determined using a slightly modified Folin-Ciocalteu assay [36]. The extract sample (150 μL) was mixed with 750 μL of Folin-Ciocalteu reagent, at room temperature, for 5 min. Then, 4 mL of 15% (w/w) sodium carbonate were added and the volume was made up to 15 mL with distilled water. The mixture was kept at room temperature, for 30 min and the absorbance was measured at 765 nm, using an UV/VIS spectrophotometer (Jasco V650, Japan). All determinations were carried out in triplicate. Caffeic acid was used as standard for the calibration curve. Total phenolic content values were expressed as milligrams of caffeic acid equivalents per gram dry extract.

Total flavonoid content of the plant extracts was determined by a colorimetric method [37]. Each 0.5 mL solution of plant extract were separately added to 1.5 mL of methanol, 0.1 mL of 10% aluminum chloride, 0.1 mL of 1 M sodium acetate and 2.8 mL of distilled water. The mixture was left at room temperature, for 30 min. The absorbance was measured at 415 nm using an UV/VIS spectrophotometer (Jasco V650, Japan). All samples were run in triplicate. Quercetin was used as standard for the calibration curve. Flavonoid content was expressed as milligrams of quercetin equivalents per gram dry extract.

### HPLC analysis

Plant extracts were analyzed on a Zorbax XDB C18 reverse phase column (column size: Φ 4.6 x 150 mm) using an Agilent 1200 HPLC system (Agilent, USA), comprising a quaternary pump, a thermostated autosampler and a diode array detector. A stock solution of plant extract (1 mg/mL) was prepared in methanol and 20 μL of sample were injected. The mobile phase consisted of phase A (2 mM sodium acetate buffer, pH 3.05) and phase B (acetonitrile). Chromatographic separation was carried out with a flow rate of 0.5 mL/min, as follows: 0–30 min, 2-20% B in A; 30–40 min, 30% B in A; 40–55 min, 2% B in A. The identification of the compounds was carried out from the retention time, at a wavelength of 270 nm. Quantification of the compounds was carried out from the peak area, in comparison with authentic standards of phenolic acids (gallic acid, chlorogenic acid, caffeic acid, p-coumaric acid, ferulic acid) and flavonoids (rutin, luteolin, quercetin, myricetin, apigenin). Three experiments were performed.

### Antioxidant activity

#### DPPH free radical-scavenging activity

The free radical-scavenging activity of the extracts was determined by a colorimetric method, using the stable DPPH radical [21]. Briefly, different concentrations (10, 100, 300, 500 and 1000 mg/L) of each ethanolic plant extract (150 μL) were mixed with 0.9 mL of 0.1 M Tris–HCl buffer, pH 7.4 and 1.5 mL DPPH methanolic solution (0.25 mM). The mixture was incubated in darkness, at room temperature, for 20 min and the absorbance was measured at 517 nm against a blank (DPPH methanolic solution) using an UV/VIS spectrophotometer (Jasco V650, Japan). The antioxidant activity was expressed as DPPH free radical scavenging percentage and was calculated using the following formula:

(1) $$\%\;\text{scavenging}\;=\;\left({\text{A}}_{\text{blank}}–\;{\text{A}}_{\text{sample}}\right)/{\text{A}}_{\text{blank}}\text{x}100$$

where A_blank_ is the absorbance of DPPH methanolic solution and A_sample_ is the absorbance of the DPPH solution after the addition of sample.

Trolox was used as control. Each experiment corresponded to triplicate samples. The concentration of sample that inhibited 50% of DPPH free radicals (IC_50_, mg/mL) was determined graphically from the linear regression curve plotted between percent of inhibition and extract concentration.

#### TEAC assay

The antioxidant capacity was measured using the method of Re et al. [20] with some modifications. Briefly, the ABTS radical cation was generated by mixing a stock solution of 7 mM 2,2’-azino-bis(3-ethyl-benzo-thiazoline-6-sulfonic acid) diammonium salt (ABTS) with 2.45 mM potassium persulfate (1:1, v/v) and incubation for 12–16 h in the dark, at room temperature, until the reaction was complete and the absorbance was stable. The absorbance of the ABTS radical solution was equilibrated to a value of 0.70 ± 0.02 at 734 nm after dilution with bidistilled water. Then, 1 mL reagent was mixed with 100 μL test sample (0.05–1 mg/mL) and the absorbance was measured after 6 min, at 734 nm, using an UV/VIS spectrophotometer (Jasco V 650, Japan). Data was recorded in triplicate. Trolox was used as a standard, in the concentration range of 0–250 μM to construct a calibration curve and the antioxidant capacity was calculated as micromoles Trolox equivalents (TE) per gram of extract.

#### ORAC assay

The ORAC assay was conducted as previously described by Ou et al. [19] with some modifications. Briefly, the reaction mixture was prepared by adding 50 μL of 0.42 μM fluorescein to 100 μL extract and 1.8 mL phosphate buffer, pH 7.3. This mixture was incubated at 37°C, for 15 min. Then, 50 μL of 640 mM 2,2’-azobis[2-methyl-propionamidin] dihydro-chloride (AAPH) was added, as a peroxyl radical generator. The samples and standard solution of Trolox were daily prepared. The intensity of relative fluorescence was monitored at every 0.004 min, for 80 min, on a Perkin Elmer LS 55 spectrometer with fluorescent filters (excitation 489 nm, emission 515 nm). All tests were run in triplicate and averaged. The results, expressed as micromoles Trolox equivalents (TE) per gram of extract, were calculated according to the following formula:

(2) $$µ\text{mol TE}\;=\;{\text{C}}_{\text{Trolox}}\cdot \;\text{k}\;\cdot \left({\text{AUC}}_{\text{sample}}–{\text{AUC}}_{\text{blank}}\right)/\left({\text{AUC}}_{\text{Trolox}}–{\text{AUC}}_{\text{blank}}\right)$$

where: C_Trolox_ is Trolox concentration, k is the sample dilution factor, AUC is the area below the fluorescence decay curve for the sample, blank, and Trolox, respectively.

### *In vitro* tests on NCTC cell culture

#### Cell culture and cytotoxicity experiment

Mouse fibroblast cells (NCTC clone 929) were grown in Minimum Essential Medium (MEM) containing 10% fetal bovine serum (FBS), 100 U/mL penicillin, 100 mg/L streptomycin and 500 mg/L neomycin. Confluent cells were trypsinised, centrifuged and subcultured in the same medium, in a humidified 5% CO_2_/95% air atmosphere, at 37°C. For experiments, cell suspensions were seeded at a density of 5x10^4^ cells/well in 24-well culture plates, allowed to adhere by culturing in MEM containing 10% FBS and incubating in a humidified 5% CO_2_ atmosphere, at 37°C, for 24 h. Then, the culture medium was replaced with the same medium, containing various concentrations of plant extract (10, 100, 300, 500 and 1000 mg/L) and cells were cultured in standard conditions, for 24 h. Cell viability was assessed by Neutral red assay and cell integrity was assessed by LDH assay. Untreated cells and cells cultured in the presence of 100 μM H_2_O_2_ served as controls. Three separate experiments were conducted.

#### Neutral red assay

The cytotoxicity of plant extracts was assessed according to Neutral red method, as previously described [38] with some modifications. Briefly, at 24 h after H_2_O_2_ treatment, the culture media were replaced with NR solution (50 mg/L) in MEM and the cells were incubated at 37°C, for 3 h. Then, fibroblasts were washed and the retained NR was dissolved using 1% (v/v) acetic acid in 50% (v/v) ethanol. The plates were incubated on a shaker for 15 min and absorbance was measured at 540 nm on a microplate reader (Sunrise Tecan, Austria). Results, expressed as mean of six determinations ± standard deviation (SD), were reported as percentage from the untreated cell control, considered as 100% viable cells.

#### LDH activity assay

LDH activity, as indicative of cell membrane damage [39], was measured using CytoTox 96 kit according to the manufacturer’s protocol. Briefly, culture medium and cell lysates collected after experiments in serum-free medium were centrifuged and an aliquot of 50 μL from supernatant was incubated with 50 μL mixed reaction solutions, at room temperature, for 30 min. The absorbance was measured at 490 nm using a 96-well plate reader (Sunrise Tecan, Austria). The intensity of the color is proportional to LDH activity. Results were calculated as follows:

(3) $$\text{LDH released}\;\left(\%\right)\;=\;{\text{LDH}}_{\text{medium}}/\;{\text{LDH}}_{\text{medium}\;+\;\text{cell lysate}}\text{x}\;100$$

where, LDH_medium_ is the LDH activity from the culture medium and LDH_medium + cell lysate_ is the LDH activity from the culture medium and cell lysate. Data were expressed as mean of six determinations ± SD.

#### H_2_O_2_-induced oxidative stress in cell culture and plant extract treatment

In order to establish the H_2_O_2_ concentration that provide recoverable cell damage (approx. 50%), cells were treated with several concentrations of H_2_O_2_ (20, 50, 100, 500 μM), for 24 h. Cell viability was assessed by Neutral red assay.

To evaluate the cytoprotective effect of plant extracts, cells were seeded in 24-well culture plates, at a density of 5x10^4^ cells/well and allowed to adhere for 24 h, by incubating in MEM containing 10% FBS, in a humidified 5% CO_2_ atmosphere, at 37°C. Two treatment groups were designed: cells simultaneously treated with plant extract and H_2_O_2_ (co-treatment) and cells treated with various concentrations of plant extracts for 24 h and then exposed to H_2_O_2_ (pre-treatment). Plates were incubated at 37°C, for 24 h and cells from both groups were analyzed for their viability by Neutral red and LDH assays, morphology by light microscopy observations and cell cycle analysis by flow-cytometry. Untreated cells and cells treated with H_2_O_2_, cultivated in the same conditions, were used as controls in this experiment. Three independent assays were carried out.

#### Light Microscopy

The cultured cells were fixed in methanol and Giemsa stained. The cultures were photographed at a Zeiss AxioStar Plus microscope equipped with a digital camera driven by AxioVision 4.6 software (Carl Zeiss, Germany).

#### Flow-cytometric analysis of DNA content and cell cycle distribution

DNA content and cell cycle distribution were assessed by staining with propidium iodide (PI), as previously described [40]. Briefly, cells were seeded onto 6-well plates, at a density of 2x10^5^ cells/well and incubated in MEM supplemented with 10% FBS, for 24 h. The cells were treated with plant extracts and/or H_2_O_2_ as indicated for co- and pre-treatment protocols. Upon treatment, cells were harvested with 0.02% trypsin and 0.2% EDTA, rinsed with PBS (5 min) and fixed in 70% ethanol, at 4°C, for 24 h. Cells washed twice with PBS (5 min) were incubated in 0.5 mL solution of 0.5 mg/mL RN-ase, at 37°C for 30 min. After rinsing, the cells were incubated in 50 mg/L PI solution in PBS, at 4°C, for 30 min and analyzed at a LSR II flow-cytometer (Becton Dickinson, USA). MODFIT™ LT 3.0 software was used to deconvolute cell DNA content histograms into cell percentage of each cell cycle phase (G0/G1, G2/M and S). Untreated cells and cells treated with H_2_O_2_ were used as controls. Three separate experiments were conducted.

### Statistical analysis

Triplicates for chemical analysis and three experiments with duplicates for cell culture studies were performed for each sample. Data were reported as mean ± SD. Pair comparison of control and each sample was carried out by t*-*test. Significant statistical differences were considered at p < 0.05 (^#^p < 0.05, ^##^p < 0.01 compared to untreated control; ^*^p < 0.05, ^**^p < 0.01 compared to H_2_O_2_-treated group).

## Conclusions

In conclusion, our data demonstrated that ethanolic extracts of *A. montana* and *A. absinthium* presented high phenolic acids and flavonoids content, a good antioxidant activity and are cytocompatible in NCTC cell line, at concentrations up to 100 mg/L and 500 mg/L, respectively. Both plant extracts possessed fibroblast protective effect against hydrogen peroxide-induced oxidative stress in cultured cells. Based on these findings, we consider that the traditional use of arnica and wormwood in skin disorders treatment could be explained by their antioxidant and cytoprotective activities. Further studies will determine which compounds from these plant extracts are responsible for their protective properties.

## Competing interests

The authors declare that they have no competing interests.

## Authors’ contributions

OC participated in measurement of radical scavenging activity, carried out data interpretation, statistical analysis and helped draft the manuscript. DC and LT carried out the *in vitro* tests and flow-cytometry on NCTC cell culture, acquisition of data and performed data analysis. AG and EU produced samples, carried out the determination of total phenolics, HPLC, measurement of radical scavenging activity and performed data analysis. LM conceived the study, participated in the design and co-ordination of the experiments, interpretation of data and drafted the manuscript. All authors read and approved the final manuscript.

## Supplementary Material

Additional file 1 **HPLC profile of *A. montana *L. (A) and *A. absinthium *L. (B) extracts.** Instrumental conditions are as described in the Experimental section. Click here for file

Additional file 2 **HPLC profile of reference standards of phenolic acids and flavonoids.** 1- gallic acid; 2- chlorogenic acid; 3- caffeic acid; 4- p-coumaric acid; 5-ferulic acid; 6-rutin, 7-myricetin, 8-luteolin, 9-quercetin; 10-apigenin. Click here for file
