# Supplementary figures and images for: Evaluation of antioxidant and cytoprotective activities of Arnica montana L. and Artemisia absinthium L. ethanolic extracts
Source: Chem Cent J. 2012 Sep 9;6:97. doi: 10.1186/1752-153X-6-97 (PMC3472325; doi:10.1186/1752-153X-6-97)

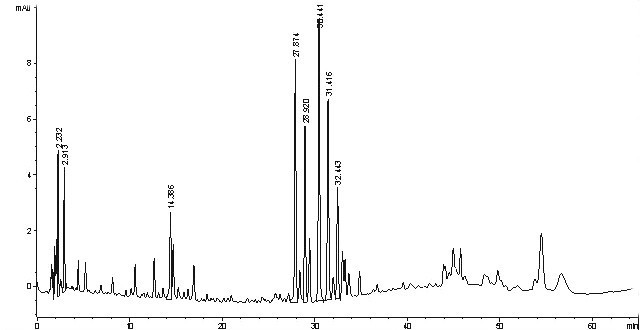


**A**


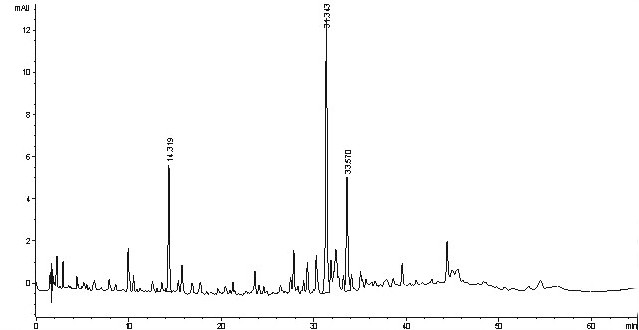


**B**

**Additional file 1**

Supplement: Additional file 1 — HPLC profile of A. montana L. (A) and A. absinthium L. (B) extracts. Instrumental conditions are as described in the Experimental section. [file 1752-153X-6-97-S1.doc]

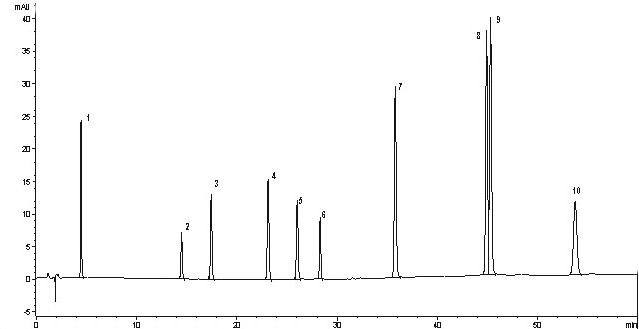


**Additional file 2**

Supplement: Additional file 2 — HPLC profile of reference standards of phenolic acids and flavonoids. 1- gallic acid; 2- chlorogenic acid; 3- caffeic acid; 4- p-coumaric acid; 5-ferulic acid; 6-rutin, 7-myricetin, 8-luteolin, 9-quercetin; 10-apigenin. [file 1752-153X-6-97-S2.doc]
